# Pain Management in Children Admitted to the Emergency Room: A Narrative Review

**DOI:** 10.3390/ph16081178

**Published:** 2023-08-18

**Authors:** Daniela Cunico, Arianna Rossi, Matteo Verdesca, Nicola Principi, Susanna Esposito

**Affiliations:** 1 Pediatric Clinic, Department of Medicine and Surgery, University of Parma, 43126 Parma, Italy; dani.cunico@gmail.com (D.C.); arianna.rossi@unipr.it (A.R.); matteo.verdesca@unipr.it (M.V.); 2 Università degli Studi di Milano, 20122 Milan, Italy; nicola.principi@unimi.it

**Keywords:** ibuprofen, NSAID, opioids, pain, paracetamol

## Abstract

Pain is a biopsychosocial experience characterized by sensory, physiological, cognitive, affective, and behavioral components. Both acute and chronic pain can have short and long-term negative effects. Unfortunately, pain treatment is often inadequate. Guidelines and recommendations for a rational approach to pediatric pain frequently differ, and this may be one of the most important reasons for the poor attention frequently paid to pain treatment in children. This narrative review discusses the present knowledge in this regard. A literature review conducted on papers produced over the last 8 years showed that although in recent years, compared to the past, much progress has been made in the treatment of pain in the context of the pediatric emergency room, there is still a lot to do. There is a need to create guidelines that outline standardized and easy-to-follow pathways for pain recognition and management, which are also flexible enough to take into account differences in different contexts both in terms of drug availability and education of staff as well as of the different complexities of patients. It is essential to guarantee an approach to pain that is as uniform as possible among the pediatric population that limits, as much as possible, the inequalities related to ethnicity and language barriers.

## 1. Introduction

According to the International Association for the Study of Pain (IASP), pain is defined as an aversive sensory and emotional experience typically caused by, or resembling that caused by, actual or potential tissue injury [1]. Pain may be acute or chronic. Acute pain is generally associated with the development of a disease, i.e., the exacerbation of an already known disease or the implementation of invasive medical procedures. Chronic pain includes recurrent and persistent pain. Recurrent pain is a pain that occurs at least three times throughout a period of 3 months, whereas persistent pain is a pain that last more than 3 months [2]. In children, pain, both acute and chronic, is very common. A school survey using a structured self-report questionnaire has shown that 60% of children and adolescents reported pain within the previous 3 months and that 21% suffered from chronic pain [3]. Moreover, a cross-sectional observational study of 26,180 patients aged from 2 to 19 years seen in primary care clinics has shown that pain was reported by the patient or caregivers in 14.9% of visits [4]. The most common causes of acute pain in children are sore throat (72%), respiratory tract infections (71%), stomachache (64%), pain associated with immunization (59%), headaches (54%), earache (54%), toothache (53%), muscle ache (47%), tension headache (39%), and postsurgical pain (39%) [5]. Children suffering from sickle cell disease, hemophilia, juvenile idiopathic arthritis, inflammatory bowel disease, hereditary angioedema, cancer, Mediterranean fever, Fabry’s disease, and Gaucher’s disease are those at increased risk of chronic pain [6].

Pain is a biopsychosocial experience characterized by sensory, physiological, cognitive, affective, and behavioral components [1]. Acute pain can have both short and long-term negative effects. Acute consequences include an increase in the child distress and the development of anxiety and fear as well as a heightening of the pain experience leading to hyperalgesia. The long-term effects of acute pain are the risk of the development of chronic pain when nerve stimulation precipitates a series of altered pain pathways, resulting in central sensitization and impaired central nervous system mechanisms [7,8]. Chronic pain can significantly weaken the physical and psychological integrity of the patient and his family. Sleep, cognitive process, brain function, mental health, and cardiovascular health can be impaired with a significant reduction in quality of life [9,10,11]. Optimal pain treatment is of extreme importance to avoid these risks. 

Unfortunately, pain treatment is often inadequate. Many children do not receive pain-relieving therapy in the emergency room. Moreover, despite the fact that it is well known that timely administration of adequate analgesic therapy positively influences the outcomes of the underlying condition and affects satisfaction of the child and his/her parents, antalgics are frequently given much later than needed [12,13]. Protocols for pain treatment specifically prepared for children may significantly reduce these limitations. Several national and international scientific institutions have prepared guidelines for a rational approach to pediatric pain [14,15,16,17,18]. However, the recommendations frequently differ, and this may be one of the most important reasons for the poor attention frequently paid to pain treatment in children. This narrative review discusses the present knowledge in this regard. We conducted a search of PubMed using these words combined with the use of Boolean operators: “pain management” OR “management of pain” AND “children”. The search was limited to published articles written in the last 8 years (from 05/2015 to 05/2023) and regarding pediatric age (0–18 years). Then, all the identified studies were evaluated by title or abstract, and very specific articles, concerning categories of patients affected by chronic pathologies were excluded. A small amount of information that was considered important was taken from sources not included in the selection. 

## 2. Assessment of Pediatric Pain

A comprehensive pain assessment is essential to guide clinical pain management. To face this problem, various rating scales for pediatric pain assessment have been prepared [1]. For preverbal and non-verbal children of any age who are unable to speak or cannot use appropriate words to express their discomfort adequately, behavioral signs and physiological changes are utilized to define the presence and severity of pain [19,20,21,22,23]. Behavioral measures that can suggest pain include facial expression, excessive crying and irritability, poor feeding, sleep disturbance, and movement of arms and legs. Evidence of an increase in heart rate, respiratory rates, and blood pressure and a reduction in oxygen saturation can favor a more reliable pain assessment [19,20,21,22,23]. All the scales specifically prepared for neonates, younger infants, or children with cognitive problems include the assessment of several of the previously reported variables. Based on the presence and intensity of each of them, a score is created that expresses the severity of the pain. However, the results should always be interpreted with caution as behavioral measures can reflect fear and anxiety rather than pain, and physiological measures may depend on stress reactions not correlated with pain [24]. Moreover, most of the more than 50 scales prepared for the assessment of pain in neonates and younger children have not been adequately validated. Those more largely used and that seem to allow the acquisition of reliable data are the Neonatal Infant Pain Scale (NIPS) [25], the Face, Legs, Activity, Cry, Consolability (FLACC) scale [26], the EValuation ENfant DOuLeur (EVENDOL) scale [27], the COMFORT scale [28], and the Neonatal Facial Coding System (NFCS) [29] scale. However, in most cases, validation has only been obtained for specific subjects and should be used only in these patients. For example, the FLACC scale has been prepared for evaluating post-operative pain in children aged from 2 months to 7 years old, whereas the EVENDOL scale has been developed for children aged from 0 to 7 years old in the emergency department and validated for use in prehospital settings. Observational scores can be also used to assess pain in toddlers and older children. In this regard, in addition to EVENDOL [27], reliable data can be collected with a modified version of the Children’s Hospital of Eastern Ontario Pain Scale (CHEOPS) that has been validated for evaluating immunization-related pain in children aged from 2 to 22 months old [30]. 

As pain is a subjective experience, in children with age and neurological development sufficient to reliably report all the information regarding the severity of the condition they are experiencing, pain should be evaluated through self-reporting rather than by proxy [31,32,33]. In children between 3 and 8 years of age, pain is quantified using photographs or drawings of faces showing an increase in distress or pain. The child is asked to choose the face that best describes his pain and record the appropriate number, from 0 to 10, that quantifies the severity. An example in this regard is given by the Wong–Baker scale [31]. From the age of 8 years, when children can understand the concept of order and number, verbal scales, numerical scales, and graphic scales, as well as the visual analogue scale, can be used. Among the scales more frequently used are the NRS-11 scale and the Color Analog Scale (CAS). The NRS-11 scale is administered verbally. The child is asked how much pain she or he has by using a number from 0 to 10, where 0 is no pain and 10 is the most or worst pain [32]. The CAS is a plastic instrument with a wedge-shaped color-gradated figure on one side, a numerical scale on the other, and a moveable slider. The child must move the slider to the place that shows how much pain he has [33]. 

The assessment of chronic pain can be significantly more difficult due to the need to evaluate not only the pain but also all the potential social, emotional, cognitive, environmental, and behavioral factors that regularly accompany chronic pain [34]. The Bath Adolescent Pain Questionnaire (BAPQ) [35], the Patient-Reported Outcome Measurement Information System (PROMIS) [36], the Pediatric Pain Interference Scale (PPIS) [37], and the Pediatric Pain Questionnaire (PPQ) [38] are the most common tools used in this regard. 

## 3. Treatment of Pediatric Pain

Generally, scales used to quantify pain severity indicate that scores ≤ 3 correspond to mild, scores of 4–6 to moderate, and scores ≥ 7 to severe pain [39,40,41,42]. In children with acute pain, mild cases should be approached with non-pharmacologic therapeutic strategies eventually associated with topical medications and/or non-opioid drugs such as paracetamol or non-steroidal anti-inflammatory drugs (NSAIDs). Children with moderate pain should receive the same treatment, although the introduction of weak oral or intranasal opioids or other analgesics should be considered [39]. In cases with severe pain, strong opioids intranasal or intravenous (IV) drugs are mandatory, but non opioid analgesics should be added because of their opioid-sparing effects [40,41,42]. In children with chronic pain, together with analgesics, psychological, physical, and occupational measures are important in order to reduce the limitations due to the underlying disease that cause pain and improve quality of life [39]. 

### 3.1. Non-Pharmacological Approach

Non-pharmacological strategies based on physical and psychological measures together with distracting activities can be useful, and they can eventually be used in association with topical or systemic pharmacological treatment to reduce pain regardless of its origin [39,43,44]. They are mainly used to reduce pain and anxiety that develop because of several routinary procedures, such as heel lancing, venipuncture, lumbar puncture, or intravenous cannulation. Psychological measures include allowing parental presence, providing reassurance, and maintaining a calm environment. Physical comfort measures based on oral stimulation with breastfeeding and pacifiers, physical contact based on skin-to-skin contact, rocking, and swaddling, are appropriate for neonates and younger infants. All the clinical manifestations of pain typically found in these subjects such as crying and increases in heart rate, respiratory rate, blood pressure, and muscle tone are significantly reduced when physical measures are used [43,44]. 

Preschool children and school-aged children and adolescents can benefit from the simultaneous use of physical measures and distracting activities. In these patients, cold or heat packs may be applied to the painful area [45,46]. Cold acts like a mild local anesthetic and can reduce swelling if applied to an acute injury. Moreover, cold packs can be associated with a vibration device in order to provide a mild noxious stimulus at one site to inhibit the pain response at a more distal site. The vibration can block the afferent pain-receptive fibers by non-noxious fibers, further blocking pain transmission [47]. Heat increases blood flow and is helpful for relieving generalized aches and stiffness. Interestingly, a recent systematic literature review investigated the effects of infant massaging on the following outcomes: pain relief, jaundice, and weight gain [48]. Although the results must be interpreted with caution, it was shown that infant massaging may be effective at relieving pain, improving jaundice, and increasing weight gain. Even if statistically significant differences were not found between all the experimental and control groups, no adverse effects of infant massaging were observed [48]. Given the dearth of research on infant massaging in the context of child health care, further research is warranted.

Distracting activities should be chosen considering the age of the patient and can be based on blowing bubbles, lighted wands, sound, and music for preschool children and books, movies, and interactive games, such as video or computer games, for the oldest. Although with differences, all these measures have been found to be effective in reducing pain, particularly during needle procedures and laceration repair procedures [49,50,51,52]. For elective needle procedures, such as blood draws, intravenous access, injections, or vaccination, overwhelming evidence now mandates that a bundle of four modalities to eliminate or decrease pain should be offered to every child every time: (1) topical anesthesia, e.g., lidocaine 4% cream, (2) comfort positioning, e.g., skin-to-skin contact for infants and not restraining children, (3) sucrose or breastfeeding for infants, and (4) age-appropriate distraction. A deferral process may include nitrous gas analgesia and sedation [53].

For chronic pain, an interdisciplinary rehabilitative approach, including physical therapy, psychological treatment, integrative mind–body techniques, and normalizing life, has been shown to be most effective [53].

Finally, because of the positive effects obtained in adults, a mind–body approach to pediatric pain management has been suggested [54]. Mindfulness, hypnosis, acupuncture, and yoga are four examples of mind–body techniques that have been proposed to reduce opioid use and improve pediatric pain management [55]. 

Failure to implement evidence-based non-pharmacological pain prevention and treatment for children in medical facilities is now considered inadmissible and a poor standard of care.

### 3.2. Pharmacological Approach

#### 3.2.1. Topical Medications

Topical anesthetics should be considered for children who are likely to require any non-emergent procedure, such as venipuncture, small abscess drainage, lumbar puncture, or wound closure [56]. For better results, it should be always considered that topical medications reduce or eliminate pain but have no effect on fear and anxiety. For this they should be always associated with non-pharmacological measures. There are several topical anesthetics that when applied on the skin can cause a reversible block in conduction along the nerve fibers for a few hours post application [57]. Examples in this regard include: (a) a eutectic mixture of local anesthetic (EMLA) cream (lidocaine 2.5% and prilocaine 2.5%), which is effective at numbing the tissue below intact skin to a depth of 6–7 mm if left on for 30–60 min, (b) LMX4, a topical liposomal 4% lidocaine cream similar to EMLA that has full effectiveness by 30 min, and (c) LET or LAT (lidocaine, epinephrine/adrenaline, and tetracaine) that works within 20–30 min when applied to open wounds [56].

#### 3.2.2. Paracetamol

Paracetamol (PC) (also known as acetaminophen or N-acetyl-p-aminophenol) is a very old drug. 

It was introduced into clinical practice more than 100 years ago [58]. Despite this, it remains the first-line treatment of fever and mild-to-moderate acute pain in patients of any age, including younger infants. It is the only recommended analgesic drug recommended in infants younger than 3 months of age [58]. The very good tolerability of the drug, particularly the lack of severe adverse events, is the main reason for PC’s continued success. The recommended doses for paracetamol vary from 10 to 15 mg/kg every 4–6 h (up to 60 mg/kg/day) [59]. The pharmacokinetic characteristics of PC vary according to age and body weight. However, the available data seem to indicate that a weight-based dosing should assure an acceptable efficacy and safety in all children.

For years, it was thought that the analgesic effect of PC depended exclusively on the inhibition of cyclooxygenase (COX) enzymes and the resulting reduction in prostaglandin (PG) synthesis [60,61]. On the contrary, recent research has shown that the analgesic activity of PC is mainly dependent on N-(4-hydroxyphenyl)-arachidonamide (AM404) [62]. This is formed in the central nervous system (CNS) from the liver metabolite of PC, p-aminophenol, under the action of the fatty acid amide hydrolase (FAAH) enzyme [63]. AM404 activates the endocannabinoid system and the transient receptor potential vanilloid-1 (TRPV-1) channel, thus modulating pain signaling [64]. Additionally, it has been suggested that PC influences voltage-gated Kv7 potassium channels and inhibits T-type Cav3.2 calcium channels, thus contributing to a reduction in pain. 

The analgesic effect of PC has been repeatedly demonstrated in several studies. Among them, of interest include those that were carried out in children with dental or tooth-extraction-related pain [65,66,67,68], in those with migraine [69], in those with pharyngotonsillitis [70], and in those with postoperative tonsillectomy pain [71,72,73]. In all these instances, the analgesic effect of PC was significantly greater than that of a placebo and noninferior to that of NSAIDs, including ibuprofen and ketoprofen [74]. Moreover, clinical studies have shown that using PC, even at low analgesic doses, allows for a significant reduction in the simultaneous administration of other non-opioid or opioid drugs, thus reducing the risk of drug-related adverse events [75].

As already highlighted, at therapeutic doses, PC is generally safe and well-tolerated with an incidence of adverse events that is not substantially different from that of ibuprofen (odds ratio (OR), 0.82; 95% confidence interval (CI), 0.60–1.12) [74,76]. Nausea, sleepiness, and constipation were the most common, occurring in about 10% of cases, especially in children receiving several doses [77]. The only severe adverse event, generally associated with the prolonged use of higher-than-recommended doses, is severe hepatoxicity that may lead to death or liver transplantation [74]. This depends on the production of a PC metabolite, N-acetyl-p-benzoquinoneimine, that causes hepatocellular damage. About 90% of administered PC is metabolized by glucuronidation and sulfation and 10% by cytochrome P450 (CYP450) with the formation of the toxic metabolite. Severe liver damage has been documented after the administration of single doses ranging from 120 to 150 mg/kg [78] or when sustained dosing (>1 day) with >90 mg/kg/day are given [79]. Little importance is, on the contrary, presently ascribed to the risk of interactions between PC and other drugs (i.e., rifampicin, phenytoin, carbamazepine, and phenobarbitone), which are metabolized in the liver and that can cause, by the induction or suppression of CYP450 activity, modifications to plasma PC concentrations, analgesic effects, and the risk of liver damage. No serious adverse drug interactions with therapeutic doses of paracetamol have been confirmed in humans.

#### 3.2.3. Non-Steroidal Anti-Inflammatory Drugs

Among this large group of drugs that include both selective and non-selective non-steroidal anti-inflammatory drugs (NSAIDs), ibuprofen (IB) is the most used to treat pain in children [74]. 

IB is a non-selective drug used to treat mild-to-moderate pain alone or in association with other analgesic measures. IB’s activity is due to the inhibition of both COX-1 and COX-2 enzymes that results in a strong interference with the conversion of arachidonic acid into prostaglandins. Prostaglandin inhibition leads to a strong reduction in inflammation and associated features with a further reduction in pain [80]. Unfortunately, these advantages are associated with the loss of the protective effects exerted by prostaglandins on the gastric mucosa, the kidney, and the cardiovascular system [81]. Adverse events such as renal failure, gastric ulcer, intestinal problems, congestive heart failure, hypertension, and thrombocytopenia have been described after IB administration as well as with all the other NSAIDs. Fortunately, severe adverse events occur almost only when IB is administered with doses significantly higher than those capable of significantly reducing pain. With the recommended dosage, only children with dehydration or those at risk of dehydration and those with varicella, pneumonia, Kawasaki’s disease, and coagulation disorders have been found, although rarely, to be at an increased risk of IB toxicity [82]. The age limits for IB use vary considerably from country to country. The British National Formulary for Children recommends IB from age 1 month. In contrast, in the USA, IB is only recommended for children aged at least 6 months [83]. The World Health Organization has authorized IB from the third month of life. However, similar weight-based doses are recommended. In children <40 kg, the dose is 5–10 mg/kg every 6 h with a maximum daily dosage of 30–40 mg/kg. Generally, a lower dose is given to infants, although these subjects could receive a higher dose, as the clearance of the drug is 98% of adult levels by 3 months of age [84]. In children >40 kg, 200–400 mg every 8 h with a maximum of 1600 mg/day are recommended [85,86,87,88]. The development of IB toxicity is uncommon for doses less than 100 mg/kg ingested over the total course of treatment. Only doses of 400 mg/kg are associated with life-threatening clinical manifestations [89,90].

Several studies carried out more than 20 years ago have reported that, at recommended doses, the analgesic activity of IB is comparable to that of paracetamol [91]. More recent evaluations, enrolling 535 participants, have indicated that for continuous pain outcomes, IB is associated with less pain from 4 to 24 h from the treatment onset (standardized mean difference (SMD), 0.20; 95% CI, 0.03–0.37; *p* = 0.02) [92]. Moreover, in children with extremity fractures, IB was found to be as effective as morphine but was tolerated significantly better. Pain scores at 30 min after administration were significantly and similarly improved by both analgesics, but the incidence of adverse events was lower in patients receiving IB [93]. Finally, the use of IB was found to produce an opioid-sparing effect. When given preoperatively, an IV dose of IB of 10 mg/kg was associated with significantly fewer doses and amounts of rescue analgesia with opioids [94]. In clinical practice, IB safety and tolerability was found to be very good and substantially not different from that of PC. The serious adverse event profile was found to be not substantially different (OR, 1.08; 95% CI, 0.87–1.33; *p* = 0.50). The coadministration of IB and PC were presumed to be superior to either agent alone, but no conclusive studies in this regard are available [95]. Finally, the risk of interactions between IB and other drugs is very low. The most frequently reported problem in this regard is kidney damage when IB is used alongside with nephrotoxic drugs such as aminoglycoside antibiotics or diuretics [92,93,94]. However, at therapeutic doses, no increased risk of renal failure in children has been reported [92,93,94].

Due to their strong anti-COX activity and the supposed risk of severe adverse event risk development, other NAIDs are not recommended to treat pain in children. Despite this, several studies regarding the use of naproxen, ketoprofen, ketorolac, and diclofenac, mainly in a perioperative setting, have been carried out. The results were generally good, showing a potential opioid-sparing effect with no severe adverse events [96,97]. Interestingly, in one study, adult patients undergoing laparoscopic cholecystectomy were randomly allocated to one of three groups on admission, depending on a prescribed post-operative analgesic regimen [98]. The patients allocated to group A received a combination of IV acetaminophen and intramuscular (IM) pethidine, the patients in group B received a combination of IV acetaminophen and IV parecoxib, and the patients in group C received IV acetaminophen monotherapy. Analgesic therapy was administered at regular intervals. Pain was evaluated utilizing the numeric rating scale (NRS) at five time points. The analgesic regimens of groups A and B (combination regimens consisting of IV acetaminophen and intramuscular pethidine and IV acetaminophen and IV parecoxib, respectively) were found to be of an equivalent efficacy (*p* = 1.000). In contrast, the patients in group C (acetaminophen monotherapy) had higher NRS scores compared to the patients in both groups A (*p* < 0.01) and B (*p* < 0.01). Although no data are available in children, this study confirms the notion of a significant opioid-sparing effect of parecoxib in postoperative pain management after laparoscopic cholecystectomy [98].

#### 3.2.4. Opioids

Opioids are a group of drugs that have several effects, including deep analgesia [99]. Naturally occurring, synthetic, or semi-synthetic opioids have been identified, depending on how they are derived. They produce effects on neurons by acting on receptors located on the membranes of neuronal cells that exist at specific sites in the central and peripheral nervous systems. Three major types of opioid receptors, mu (µ), delta (δ), and kappa (κ), have been identified. Although with differences, all three receptors produce analgesia when an opioid binds to them [96]. Receptor agonists produce analgesia by indirectly stimulating descending inhibitory pathways with the effect of an activation of descending inhibitory neurons [100].

Opioids are classified based on the characteristics of how they bind to opioid receptors [101]. Those that strongly bond to receptors, primarily mu (µ) receptors, in the brain and spinal cord are defined as strong agonists. They are mainly used to treat severe pain. Examples include morphine, fentanyl, meperidine, and methadone. Mild-to-moderate agonists are, on the contrary, those opioids that bind to receptors to a lesser degree than strong agonists and are used to treat moderate pain. Among them are codeine, tramadol, hydrocodone, and oxycodone [101]. Constipation, drowsiness, itchiness, nausea, and vomiting are common opioid-associated adverse events, especially when they are chronically administered. Some children may require additional medications to help control these side effects if they become intolerable. In some cases, opioids can also alter a child’s mood by making them feel euphoric or teary. However, the most important clinical problems, generally when high doses are used, are hypotension and respiratory depression that can be severe enough to cause death. For this reason, children receiving opioids, especially strong agonists given IV for post-surgery analgesia, must be carefully monitored, and a dose of naloxone, a very strong opioid antagonist, must be always available in order to quickly reverse the effects of the opioids [102]. In the following sections, details of the most commonly used opioids for the treatment of pediatric pain are reported.

##### Morphine

Morphine remains the most used opioid in children with acute severe pain and in children with postoperative pain. When administered in proper doses, it can significantly reduce the pain score and the consumption of rescue analgesics with an efficacy not substantially different or even greater than that of codeine, fentanyl, bupivacaine, or ketorolac [103,104,105]. Moreover, although several studies have shown that morphine has a side effect profile quite similar to that of most opioids, this drug is perceived by most parents and several health care providers as being potentially more at risk of severe adverse events than other opioids. This explains why, for several years, morphine has been substituted by other opioids, mainly codeine, for the treatment of severe pain in children [106]. Only in recent years, when the risk of poor efficacy or severe adverse effects from codeine use was clearly evidenced, morphine administration for pain treatment has increased [105].

##### Codeine

Codeine (CD) is a pro-drug. It is relatively ineffective as an analgesic and can become effective only when it is metabolized to morphine by the cytochrome P450 isoenzyme CYP2D6 [107].

Polymorphisms occurring in CYP2D6 can significantly influence CD metabolism and morphine formation. Four different categories of phenotypes have been described: ultra-rapid metabolizers, extensive metabolizers, intermediate metabolizers, and poor metabolizers. Individuals classified as poor metabolizers have a poor analgesic effect from standard CD doses. On the contrary, ultra-rapid metabolizers that can develop up to 45-fold higher concentrations of morphine have a very relevant analgesic effect from CD administration but are at a very high risk of developing all the severe adverse events commonly ascribed to opioids [108,109]. Whereas poor metabolizers are about 5–10% of the population [110], ultra-rapid metabolizers are found in about 3–10% in Europe [111] and between 10% and 30% in Arabian and northeast African countries [112]. Moreover, the final effect of codeine administration can be further influenced by additional polymorphisms affecting morphine metabolism, blood–brain barrier transition, or opioid and opiate receptor kinetics [107,108,109]. Lacking genetic studies, the metabolism and analgesic effect of codeine as well as the risk of adverse events remain unpredictable. On the other hand, genotyping of the CYP2D6 enzyme is not routinely conducted in the clinical setting as it is expensive and not widely available outside of research facilities. This means that the results of CD administration remain totally unpredictable.

CD, alone or in combination with PC or IB, has been used to treat pain in children for many years because it is available in both liquid and tablet forms, it is relatively inexpensive, and it is initially considered safe, well-tolerated, and generally effective, although with differences between children with similar clinical conditions. [113]. In recent years, however, the knowledge that the final effects of CD could significantly vary, the availability of alternative drugs with similar analgesic effects, and the reports of several cases of respiratory depression and death in children that had received standard doses of codeine have led several scientific institutions worldwide to contraindicate the use of CD in children [114].

##### Tramadol

Tramadol is a weak opioid that has replaced CD in the management protocols of pediatric pain since this drug stopped being recommended by the EMA [115] and FDA [116].

However, with time, recommendations for the use of this drug in children have significantly varied. In Europe, tramadol is approved for the treatment of moderate-to-severe pain in children over 1–3 years of age, depending on the country, with restrictions for children after tonsillectomy or adenoidectomy and in those with significant respiratory problems [117]. In the USA, where the off-label use of the drug remains significant [118], it is officially not recommended to use tramadol in children <12 years old and in adolescents <18 years old after ear, nose, and throat surgery. Moreover, it is suggested to pay attention to the use of tramadol in subjects between 12 and 18 years who are obese or have conditions that may increase the risk of serious breathing problems [119].

Restrictions to tramadol use are strictly dependent on the potential development of severe adverse events following drug administration. Similar to codeine, tramadol is primarily metabolized by CYP2D6 [117,118,119]. This means that in CYP2D6 ultrarapid metabolizers, therapeutic doses of tramadol may lead to very high concentrations of O-desmethyltramadol, the main active metabolite of tramadol. A very relevant alteration to the release of nociceptive neurotransmitters occurs, and this is followed by the development of all the adverse events associated with a massive opioid administration. Sedation, nausea, vomiting, dizziness, and constipation have been frequently described in children receiving tramadol. Moreover, severe respiratory compromise leading to death has been reported, although rarely [120]. Tramadol is also metabolized by CYP3A4 and CYP2B6 with the formation of N-desmethyltramadol. This leads to the inhibition of the reuptake of the neurotransmitter and further potentiates the analgesic effect. However, in poor CYP2D6 metabolizers, most of the administered tramadol is metabolized through the second pathway, and serotonin syndrome can occur [121]. The analgesic activity of tramadol has been largely documented in adults with acute or chronic pain that had received the drug through IV, IM, subcutaneous, oral, and sublingual routes [122]. Studies in children have generally been carried out in the management of acute pain or in a perioperative setting. Data regarding its chronic administration are lacking [123]. The results of the available studies have shown that single or repeated (every 6–8 h) 1–3 mg/kg (maximum 400 mg daily) IV, oral, or sublingual doses are generally effective in reducing or suppressing severe pain. Moreover, its topic administration has been found to be effective in controlling postoperative pain in children after tonsillectomy [124,125,126]. More details about tramadol’s efficacy were obtained by pooling the results of 20 randomized controlled trials involving 1170 patients who had received the drug for postoperative pain treatment [127]. In this meta-analysis, it was shown that children receiving tramadol needed less rescue analgesia in the postoperative care unit than those given a placebo (relative risk (RR), 0.40; 95% CI, 0.20–0.78). On the contrary, the potential superiority of tramadol over other opioids, including morphine, remains uncertain. Finally, as adverse events were not adequately monitored in most of the studies, the risk related to tramadol administration could not be calculated. In conclusion, several aspects of tramadol use in children need further investigations, and this may explain why American health authorities do not currently recommend its use in younger children.

##### Fentanyl

Fentanyl (FE) is a synthetic opioid that is 75–200 times more potent than morphine, although it is generally better-tolerated [128].

It has less hypotensive effects and is safer in patients with hyperactive airway disease because its use is not associated with significant histamine release. FE is available in several formulations with different strengths. In children, the most common route of administration is the intranasal route. Transdermal and IV FE are occasionally used in the treatment of chronic pain and very severe acute pain, respectively. An intranasal FE dose of 1.5 µg/kg (maximum 100 µg), using the standard fentanyl solution of 50 µg/mL, is generally sufficient to face mild-to-severe acute pain in children >1 year [128]. This allows for achieving therapeutic levels within 2 min of administration with a duration of action of about 30–60 min. A second dose can be given after 5–10 min if required.

The efficacy of intranasal FE has been demonstrated in several studies enrolling children with burns dressings, acute long bone fractures, abdominal pain, and postoperative analgesia [129,130,131]. When compared to other pain treatments, intranasal FE was found to be at least equally effective. In studies in which intranasal FE was compared to morphine, it was shown that intranasal FE was as effective as IV morphine and induced a more rapid reduction in pain than IM morphine. Generally, standard doses of intranasal FE were found to be safe and well-tolerated. Transient hypoxia was reported in 13% of patients, transient hypotension in 8% of patients, and drowsiness in 42% of patients [132].

Transdermal FE patches can be used to treat severe long-term pain in children from 2 years of age who have chronic pain, those who have already been receiving at least 30 mg of oral morphine equivalents per day, and those who have developed a tolerance to other opioids. The lowest-strength patch capable of reducing pain should be initially used, and the patch should be replaced every 3 days [133]. However, because a wide range of plasma concentrations have been observed after its transdermal administration, the dose should be re-assessed regularly during treatment, and the lowest effective dose should be used. A blood concentration of 0.6 ng/mL to 3.0 ng/mL is appropriate for analgesia. The risk of severe adverse events in monitored patients is low, although nausea (10–90%), vomiting (10–90%), and constipation (50%) are frequently described [134].

IV FE administration by slow IV push (over 1.2 min) can be used to treat very severe pain [129,130,131]. In children <6 years, IV FE is given at 0.3–1.5 µg/kg/dose. In children aged ≥6 years, it is administered at 1–5 µg/kg/dose. Further doses can be given every 1–2 h. Monitoring of respiratory conditions is mandatory, and naloxone must be always available if severe respiratory depression occurs.

As FE is hepatically metabolized via the CYP450 enzyme system, specifically CYP3A4, it should not be used concomitantly with drugs such as macrolide antibiotics or azole-antifungal agents that inhibit these enzymes. In this case, a significant increase in FE concentrations with an increased risk of opioid-related severe adverse events, including respiratory depression, can occur [135,136]. Attention should also be paid to patients that receive CYP3A4 inducers, such as carbamazepine. In this case, the analgesic effect of FE can be significantly reduced [137].

### 3.3. Other Analgesic Agents

#### 3.3.1. Nitrous Oxide

Nitrous oxide (NO) is an odorless and colorless gas that has been used for years for general anesthesia with other anesthetic agents [138]. It also possesses analgesic properties, acting as a partial agonist at the opioid receptors. NO is given by inhalation through demand systems in older children or by flow-through systems in younger children. It is mixed with O2 (30–50%) to avoid hypoxemia, and it is given for 15–30 min. The analgesic effect is reached within 2–5 min and persists for the same amount of time. NO is used for the short-term treatment of mild-to-moderate pain or as a bridge to the IV administration of more effective anesthetics. A systematic review of 30 clinical trials performed in children [138] showed that: (1) NO was more effective than subcutaneous lidocaine and oral midazolam and as effective as IV ketamine for pain during laceration repair; (2) the efficacy of NO for pain during fracture reduction was quite similar to that of IM meperidine plus promethazine, regional anesthesia, and IV ketamine plus midazolam; (3) NO was more effective than O2 for pain during lumbar puncture; (4) NO was comparable to oral midazolam for pain during urethral catheterization; and (5) a treatment of NO plus local anesthetics was more effective than NO or local anesthetics alone for pain during intramuscular injection.

Despite being generally well-tolerated and safe, NO inhalation can be accompanied by lightheadedness, headache, dizziness, confusion, nausea, and vomiting as well as euphoria (laughing gas) [139,140,141]. NO is contraindicated in cases of pneumothorax, middle ear obstruction, intestinal obstruction, or recent eye surgery because it causes the expansion of gas-filled structures when inhaled. NO is also contraindicated in patients with intracranial hypertension or patients with head trauma because it results in an increase in cerebral blood flow, further increasing intracranial pressure [139,140,141].

#### 3.3.2. Ketamine

Ketamine is an N-methyl-D-aspartate (NMDA) receptor antagonist that is mainly used as an anesthetic. At a dose of 1.0 to 1.5 mg/kg IV or 3 to 4 mg/kg IM, ketamine induces the so-called dissociative anesthesia, a condition in which pain relief, sedation, and amnesia are obtained without significant interference with cardiovascular and respiratory systems [142]. At lower, non-dissociative doses (0.1–0.3 mg/kg), ketamine was supposed to be used as an analgesic in the emergency setting as a single agent or as an adjunct to opioids. Several clinical trials carried out in adults have confirmed this supposition, despite the fact that low-dose ketamine was associated with a higher risk of neurological (RR, 2.17; 95% CI, 1.37–3.42; *p* < 0.001) and psychological events (RR, 13.86; 95% CI, 4.85–39.58; *p* < 0.001) compared to opioids [143].

Ketamine is an analgesic and a subdissociative anesthetic. Its potential use in children was shown in a recent systematic review by Alanazi [144]. This author analyzed the results of four double-blind, randomized, controlled pediatric trials, in which ketamine was compared to morphine and fentanyl in children admitted to the emergency foom for trauma and injuries and treated for acute and severe pain. In two studies, ketamine was administered intranasally (1 mg/kg and 1.5 mg/kg), in one study by IM (0.2 mg/kg), and in the last study by IV (0.2 mg/kg). In all the studies, the results showed that ketamine was non-inferior to opioids in producing pain-reduction outcomes. On the basis of these findings, the author concluded that ketamine could be considered a suitable alternative to opioids for the management of acute and severe pain in children [144]. Reports indicated that ketamine use was accompanied by a more frequent development of adverse events such as dizziness, sleepiness, an unpleased taste in the mouth, visual disturbances, itchy nose, sedation, and amnesia [136]. Fortunately, these were mild and transient and did not impede the treatment duration. However, further studies are needed for a more precise definition of which route of administration and which dosage to allow to provide an adequate analgesic effect and obtain the best results with the lowest risk of adverse events.

#### 3.3.3. Dexmedetomidine

Dexmedetomidine is a highly selective α2-adrenoceptor agonist that has been largely used in adults as an anesthetic, mainly because it provides sedation without causing respiratory depression, although it can depress the cardiovascular system [145]. Dexmedetomidine is an alpha-2-adrenergic receptor agonist that produces analgesia by silencing the central sympathetic response. In the past it was used in sedation, whereas today it may also be used as an analgesic adjuvant, reducing the consumption of opioids and their side effects. Dexmedetomidine has fewer respiratory side effects than opioids, particularly apnea, and can be administered via a variety of routes including oral, nasal, and IV [145]. This drug is not approved for use in children. Despite this, the worldwide use of dexmedetomidine for pediatric anesthesia and analgesia is still increasing [146]. Some studies have measured its perioperative analgesic effect in children, infants, and neonates, showing that the intraoperative use of this drug (0.5–1 μg/kg/h) is associated with a reduced postoperative opioid administration (RR, 0.31; 95% CI, 0.17–0.59) and decreased pain intensity (SMD, −1.18; 95% CI, −1.88–−0.48), although no effect upon postoperative nausea and vomiting could be demonstrated (RR, 0.67; 95% CI, 0.41–1.08) [147]. However, further studies in children are needed in order to precisely define the most effective and safe drug dosage for any pediatric age and to obtain official authorization for pediatric use by health authorities [148,149].

## 4. Conclusions

Although in recent years, compared to the past, much progress has been made in the treatment of pain in the context of the pediatric emergency room, there is still a lot of work to carry out due to the complexity of pediatric patients. Several studies have shown that very often there is inadequate pain treatment in children, despite the fact that there are enough tools, both pharmacological and non-pharmacological, available to professionals. Furthermore, the importance of an adequate treatment of pain in terms of immediate but also future wellbeing and on the neurodevelopment of the patient has been demonstrated. Table 1 summarized the main drugs used for pain treatment and their dosage regimes in pediatric age.

There are international differences regarding the treatment of pain, and in Europe there are also differences between countries and regions. There is a need to create guidelines that outline standardized and easy-to-follow pathways for pain recognition and pain management that are also flexible enough to take into account differences in different contexts, both in terms of drug availability and the education of staff and also of the different complexities of patients. It is essential to guarantee an approach to pain that is as uniform as possible among the pediatric population that prevents oligoanalgesia and limits the inequalities related to ethnicity and language barriers as much as possible. In addition, further studies should focus on predictors of the response and incidence of toxicity for each of therapeutic modality in order to optimally use the different therapeutic options, especially in emergency settings. Among these predictors, genetic predispositions to toxicities as well as considering the difficulties in preemptively having this information in emergency settings should be considered.

## Figures and Tables

**Table 1:** Frequently used drugs for pain treatment and their dosage regimes in pediatric age.

Drug	Dosage
Paracetamol (acetaminophen)	10–15 mg/kg, Up to 4 times a day
Ibuprofen	5–10 mg/kg orally 6–8 hourly to a maximum of 500 mg/day
Codeine	0.5–1 mg/kg orally 6–12 hourly
Morphine	Calculate exact dose based on the weight of the child Oral: 0.2–0.4 mg/kg 4–6 hourly; increase if necessary for severe pain Intramuscular: 0.1–0.2 mg/kg 4–6 hourly Intravenous bolus: 0.05–0.1 mg/kg 4–6 hourly (give slowly) Intravenous infusion: 0.005–0.01 mg/kg/h (in neonates, only 0.002–0.003)
Ketamine	0.04 mg/kg/h–0.15 mg/kg/h i.v./s.c. (titrated to effect: usually maximum 0.3 mg/kg/h–0.6 mg/kg/h) OR 0.2 mg/kg/dose–0.4 mg/kg/dose orally t.i.d., q.i.d., and p.r.n.
Tramadol	1 mg/kg–2 mg/kg 4–6 hourly (max. of 8 mg/kg/day)

## Data Availability

Not applicable.

## References

[1] Raja S.N., Carr D.B., Cohen M., Finnerup N.B., Flor H., Gibson S., Keefeh F.J., Mogili J.S., Ringkampj M., Slukak K.A. (2020). The revised International Association for the Study of Pain definition of pain: Concepts, challenges, and compromises. Pain.

[2] Williams A.C.C., Craig K.D. (2016). Updating the definition of pain. Pain.

[3] van der Venne P., Mürner-Lavanchy I., Höper S., Koenig J., Kaess M. (2023). Physiological response to pain in female adolescents with nonsuicidal self-injury as a function of severity. J. Affect Disord..

[4] Grout R.W., Thompson-Fleming R., Carroll A.E., Downs S.M. (2018). Prevalence of pain reports in pediatric primary care and association with demographics, body mass index, and exam findings: A cross-sectional study. BMC Pediatr..

[5] GSK Consumer Healthcare Global Pain Index Report 4th Edition—2020. https://www.gsk.com/media/6351/2020-global-plain-index-report.pdf.

[6] Krauss B.S., Calligaris L., Green S.M., Barbi E. (2016). Current concepts in management of pain in children in the emergency department. Lancet.

[7] Ali S., Rajagopal M., Stinson J., Ma K., Vandermeer B., Felkar B., Schreiner K., Proctor A., Plume J., Hartling L. (2022). Virtual reality-based distraction for intravenous insertion-related distress in children: A study protocol for a randomised controlled trial. BMJ Open.

[8] Young V.B. (2017). Effective Management of Pain and Anxiety for the Pediatric Patient in the Emergency Department. Crit. Care Nurs. Clin. N. Am..

[9] Haraldstad K., Sørum R., Eide H., Natvig G.K., Helseth S. (2011). Pain in children and adolescents: Prevalence, impact on daily life, and parents’ perception, a school survey. Scand. J. Caring Sci..

[10] Huguet A., Miró J. (2008). The severity of chronic pediatric pain: An epidemiological study. J. Pain.

[11] Luntamo T., Sourander A., Santalahti P., Aromaa M., Helenius H. (2012). Prevalence changes of pain, sleep problems and fatigue among 8-year-old children: Years 1989, 1999, and 2005. J. Pediatr. Psychol..

[12] Benini F., Congedi S., Rossin S., Pennella A. (2018). The PIPER WEEKEND study. Children’s and adults satisfaction regarding paediatric pain in Italian Emergency Department. Ann. Ist. Super. Sanita.

[13] Benini F., Piga S., Zangardi T., Messi G., Tomasello C., Pirozzi N., Cuttini M., PIPER Study Group (2016). Nationwide study of headache pain in Italy shows that pain assessment is still inadequate in paediatric emergency care. Acta Paediatr..

[14] Gai N., Naser B., Hanley J., Peliowski A., Hayes J., Aoyama K. (2020). A practical guide to acute pain management in children. J. Anesth..

[15] Andersson V., Bergman S., Henoch I., Simonsson H., Ahlberg K. (2022). Pain and pain management in children and adolescents receiving hospital care: A cross-sectional study from Sweden. BMC Pediatr..

[16] World Health Organization WHO Guidelines on the Management of Chronic Pain in Children Provide Recommended Physical, Psychological and Pharmacological Interventions for Pain Relief in Children Aged 0–19 Years. https://www.who.int/who-revision-of-pain-management-guidelines.

[17] British Pain Society Core Standards for Pain Management Services in the U.K. https://www.britishpainsociety.org/static/uploads/resources/files/FPM-Core-Standards-2021.pdf.

[18] Burlo F., Zanon D., Minghetti P., Taucar V., Benericetti G., Bennati G., Barbi E., De Zen L. (2023). Pediatricians’ awareness of galenic drugs for children with special needs: A regional survey. Ital. J. Pediatr..

[19] Johnston C.C., Stevens B.J., Boyer K., Porter F.L., Schechter N.L., Berde C.B., Yaster M. (2003). Development of psychologic responses to pain and assessment of pain in infants and toddlers. Pain in Infants, Children and Adolescents.

[20] Liebelt E.L. (2000). Assessing children’s pain in the emergency department. Clin. Pediatr. Emerg. Med..

[21] Drendel A.L., Kelly B.T., Ali S. (2011). Pain assessment for children: Overcoming challenges and optimizing care. Pediatr. Emerg. Care.

[22] Chiaretti A., Pierri F., Valentini P., Russo I., Gargiullo L., Riccardi R. (2013). Current practice and recent advances in pediatric pain management. Eur. Rev. Med. Pharmacol. Sci..

[23] Bauman B.H., McManus J.G. (2005). Pediatric pain management in the emergency department. Emerg. Med. Clin. N. Am..

[24] Ali S., Drendel A.L. (2023). Responsible and safe use of opioids in children and adolescents in the emergency department. Pediatr. Emerg. Med. Pract..

[25] Manworren R.C., Hynan L.S. (2003). Clinical validation of FLACC: Preverbal patient pain scale. Pediatr. Nurs..

[26] Fournier-Charrière E., Tourniaire B., Carbajal R., Cimerman P., Lassauge F., Ricard C., Reiter F., Turquin P., Lombart B., Letierce A. (2012). EVENDOL, a new behavioral pain scale for children ages 0 to 7 years in the emergency department: Design and validation. Pain.

[27] Abou Elella R., Adalaty H., Koay Y.N., Mokrusova P., Theresa M., Male B., Francis B., Jarrab C., Al Wadai A. (2015). The efficacy of the COMFORT score and pain management protocol in ventilated pediatric patients following cardiac surgery. Int. J. Pediatr. Adolesc. Med..

[28] Peters J.W., Koot H.M., Grunau R.E., de Boer J., van Druenen M.J., Tibboel D., Duivenvoorden H.J. (2003). Neonatal Facial Coding System for assessing postoperative pain in infants: Item reduction is valid and feasible. Clin. J. Pain.

[29] Crellin D.J., Babl F.E., Santamaria N., Harrison D. (2018). A systematic review of the psychometric properties of the Modified Behavioral Pain Scale (MBPS). J. Pediatr. Nurs..

[30] Howard R.F. (2003). Current status of pain management in children. JAMA.

[31] Baeyer C.L., Spagrud L.J., McCormick J.C., Choo E., Neville K., Connelly M.A. (2009). Three new datasets supporting use of the Numerical Rating Scale (NRS-11) for children’s self-reports of pain intensity. Pain.

[32] Tsze D.S., von Baeyer C.L., Bulloch B., Dayan P.S. (2013). Validation of self-report pain scales in children. Pediatrics.

[33] Dansie E.J., Turk D.C. (2013). Assessment of patients with chronic pain. Br. J. Anaesth..

[34] Goddard J.M., Robinson J., Hiscock R. (2021). Routine use of the Bath Adolescent Pain Questionnaire in a paediatric pain clinic. Br. J. Pain.

[35] Curtis S., Brandow A.M. (2017). Responsiveness of Patient-Reported Outcome Measurement Information System (PROMIS) pain domains and disease-specific patient-reported outcome measures in children and adults with sickle cell disease. Hematol. Am. Soc. Hematol. Educ. Program..

[36] Fussner L.M., Black W.R., Lynch-Jordan A., Morgan E.M., Ting T.V., Kashikar-Zuck S. (2019). Utility of the PROMIS Pediatric Pain Interference Scale in Juvenile Fibromyalgia. J. Pediatr. Psychol..

[37] Lootens C.C., Rapoff M.A. (2011). Measures of pediatric pain: 21-numbered circle Visual Analog Scale (VAS), E-Ouch Electronic Pain Diary, Oucher, Pain Behavior Observation Method, Pediatric Pain Assessment Tool (PPAT), and Pediatric Pain Questionnaire (PPQ). Arthritis Care Res..

[38] O’Donnell F.T., Rosen K.R. (2014). Pediatric pain management: A review. Mo. Med..

[39] Murphy A., O’Sullivan R., Wakai A., Grant T.S., Barrett M.J., Cronin J., Grant T.S., Barrett M.J., Cronin J., McCoy S.C. (2014). Intranasal fentanyl for the management of acute pain in children. Cochrane Database Syst. Rev..

[40] Murphy A.P., Hughes M., McCoy S., Crispino G., Wakai A., O’Sullivan R. (2017). Intranasal fentanyl for the prehospital management of acute pain in children. Eur. J. Emerg. Med..

[41] Fowler M., Ali S., Gouin S., Drendel A.L., Poonai N., Yaskina M., Sivakumar M., Jun E., Dong K., Pediatric Emergency Research Canada (2020). Knowledge, attitudes and practices of Canadian pediatric emergency physicians regarding short-term opioid use: A descriptive, cross-sectional survey. CMAJ Open.

[42] Marín Gabriel M.Á., del Rey Hurtado de Mendoza B., Jiménez Figueroa L., Medina V., Iglesias Fernández B., Vázquez Rodríguez M., Escudero Huedo V., Medina Malagón L. (2013). Analgesia with breastfeeding in addition to skin-to-skin contact during heel prick. Arch. Dis. Child. Fetal Neonatal. Ed..

[43] Shah P.S., Herbozo C., Aliwalas L.L., Shah V.S. (2012). Breastfeeding or breast milk for procedural pain in neonates. Cochrane Database Syst. Rev..

[44] Ediriweera Y., Banks J., Hall L.M., Heal C. (2022). A study protocol for a randomised controlled trial of ice to reduce the pain of immunisation: The ICE trial. Aust. J. Gen. Pract..

[45] Lane E., Latham T. (2009). Managing pain using heat and cold therapy. Paediatr. Nurs..

[46] Shaefer J.R., Lee S.J., Anderson N.K. (2017). A Vibration Device to Control Injection Discomfort. Compend. Contin. Educ. Dent..

[47] Mrljak R., Arnsteg Danielsson A., Hedov G., Garmy P. (2022). Effects of Infant Massage: A Systematic Review. Int. J. Environ. Res. Public. Health.

[48] Bailey B., Trottier E.D. (2016). Managing Pediatric Pain in the Emergency Department. Paediatr. Drugs.

[49] Cozzi G., Lucarelli A., Borrometi F., Corsini I., Passone E., Pusceddu S., Morabito G., Barbi E., Benini F. (2021). How to recognize and manage psychosomatic pain in the pediatric emergency department. Ital. J. Pediatr..

[50] Goldman R.D., Behboudi A. (2021). Pilot Randomized Controlled Trial of Virtual Reality vs. Standard-of-Care During Pediatric Laceration Repair. J. Child. Adolesc. Trauma.

[51] Odell S., Logan D.E. (2013). Pediatric pain management: The multidisciplinary approach. J. Pain. Res..

[52] Friedrichsdorf S.J., Goubert L. (2019). Pediatric pain treatment and prevention for hospitalized children. Pain Rep..

[53] Thompson T., Terhune D.B., Oram C., Sharangparni J., Rouf R., Solmi M., Veronese N., Stubbs B. (2019). The effectiveness of hypnosis for pain relief: A systematic review and meta-analysis of 85 controlled experimental trials. Neurosci. Biobehav. Rev..

[54] Brown M.L., Rojas E., Gouda S. (2017). A Mind-Body Approach to Pediatric Pain Management. Children.

[55] Ruest S., Anderson A. (2016). Management of acute pediatric pain in the emergency department. Curr. Opin. Pediatr..

[56] Ali S., McGrath T., Drendel A.L. (2016). An Evidence-Based Approach to Minimizing Acute Procedural Pain in the Emergency Department and Beyond. Pediatr. Emerg. Care.

[57] Prescott L.F. (2000). Paracetamol: Past, present, and future. Am. J. Ther..

[58] Zempsky W., Bell J., Mossali V.M., Kachroo P., Siddiqui K. (2023). Common Selfcare Indications of Pain Medications in Children. Paediatr. Drugs.

[59] Graham G.G., Scott K.F. (2005). Mechanism of action of paracetamol. Am. J. Ther..

[60] Chiappini E., Venturini E., Principi N., Longhi R., Tovo P.A., Becherucci P., Bonsignori F., Esposito S., Festini F., Galli L. (2012). Update of the 2009 Italian Pediatric Society Guidelines about management of fever in children. Clin. Ther..

[61] Ouellet M., Percival M.D. (2001). Mechanism of acetaminophen inhibition of cyclooxygenase isoforms. Arch. Biochem. Biophys..

[62] Sharma C.V., Long J.H., Shah S., Rahman J., Perrett D., Ayoub S.S., Meht V. (2017). First evidence of the conversion of paracetamol to AM404 in human cerebrospinal fluid. J. Pain. Res..

[63] Mallet C., Desmeules J., Pegahi R., Eschalier A. (2023). An Updated Review on the Metabolite (AM404)-Mediated Central Mechanism of Action of Paracetamol (Acetaminophen): Experimental Evidence and Potential Clinical Impact. J. Pain Res..

[64] Baillargeau C., Lopez-Cazaux S., Charles H., Ordureau A., DajeanTrutaud S., Prud’homme T., Hyon I., Soueidan A., Alliot-Licht B., Renard E. (2020). Post-operative discomforts in children after extraction of primary teeth. Clin. Exp. Dent. Res..

[65] Fux-Noy A., Bendahan Y., Ungar E., Shmueli A., Halperson E., Ram D., Moskovitz M. (2020). Does preoperative paracetamol reduce pain after dental treatment? A randomized controlled doubleblind study. Quintessence Int..

[66] Raslan N., Zouzou T. (2021). Comparison of preemptive ibuprofen, acetaminophen, and placebo administration in reducing peri- and postoperative pain in primary tooth extraction: A randomized clinical trial. Clin. Exp. Dent. Res..

[67] Santos P.S., Massignan C., de Oliveira E.V., Miranda Santana C., Bolan M., Cardoso M. (2020). Does the pre-emptive administration of paracetamol or ibuprofen reduce trans- and post-operative pain in primary molar extraction? A randomized placebo-controlled clinical trial. Int. J. Paediatr. Dent..

[68] Wang G., Tan T., Liu Y., Hong P. (2020). Drugs for acute attack of pediatric migraine: A network meta-analysis of randomized controlled trials. Clin. Neurol. Neurosurg..

[69] Ruperto N., Carozzino L., Jamone R., Freschi F., Picollo G., Zera M., Della Casa Alberighi O., Salvatori E., Del Vecchio A., Dionisio P. (2011). A randomized, double-blind, placebo-controlled trial of paracetamol and ketoprofren lysine salt for pain control in children with pharyngotonsillitis cared by family pediatricians. Ital. J. Pediatr..

[70] Mahgoobifard M., Mirmesdagh Y., Imani F., Najaf A., Nataj-Majd M. (2014). The analgesic efcacy of preoperative oral Ibuprofen and acetaminophen in children undergoing adenotonsillectomy: A randomized clinical trial. Anesth. Pain. Med..

[71] Mirashraf F., Tavakolnejad F., Amirzargar B., Abasi A., Amali A. (2021). Efect of paracetamol versus ibuprofen in adenotonsillectomy. Iran. J. Otorhinolaryngol..

[72] Jotić A., Savić Vujović K., Milovanović J., Vujović A., Radin Z., Milić N., Vučković S., Medić B., Prostran M. (2019). Pain management after surgical tonsillectomy: Is there a favorable analgesic?. Ear Nose Throat J..

[73] Chiappini E., Venturini E., Remaschi G., Principi N., Longhi R., Tovo P.A., Becherucci P., Bonsignori F., Esposito S., Festini F. (2017). 2016 Update of the Italian Pediatric Society Guidelines for Management of Fever in Children. J. Pediatr..

[74] Mencía S., Alonso C., Pallás-Alonso C., López-Herce J. (2022). Maternal and Child Health and Development Network Ii Samid Ii. Evaluation and Treatment of Pain in Fetuses, Neonates and Children. Children.

[75] Pierce C.A., Voss B. (2010). Efficacy and safety of ibuprofen and acetaminophen in children and adults: A meta-analysis and qualitative review. Ann. Pharmacother..

[76] Clark E., Plint A.C., Correll R., Gaboury I., Passi B. (2007). A randomized, controlled trial of acetaminophen, ibuprofen, and codeine for acute pain relief in children with musculoskeletal trauma. Pediatrics.

[77] American Academy of Pediatrics (2001). Acetaminophen toxicity in children. Pediatrics.

[78] Shivbalan S., Sathiyasekeran M., Thomas K. (2010). Therapeutic misadventure with paracetamol in children. Ind. J. Pharmacol..

[79] Simmons D.L., Botting R.M., Hla T. (2004). Cyclooxygenase isozymes: The biology of prostaglandin synthesis and inhibition. Pharmacol. Rev..

[80] Leroy S., Mosca A., Landre-Peigne C., Cosson M.A., Pons G. (2007). Ibuprofen in childhood: Evidence-based review of efficacy and safety. Arch. Pediatr..

[81] de Martino M., Chiarugi A., Boner A., Montini G., de’ Angelis G.L. (2017). Working Towards an Appropriate Use of Ibuprofen in Children: An Evidence-Based Appraisal. Drugs.

[82] Tan E., Braithwaite I., McKinlay C., Riley J., Hoare K., Okesene-Gafa K., Semprini A., Sheridan N., Grant C., Johnson D. (2020). Randomised controlled trial of paracetamol or ibuprofen, as required for fever and pain in the first year of life, for prevention of asthma at age 6 years: Paracetamol or ibuprofen in the primary prevention of asthma in Tamariki (PIPPA Tamariki) protocol. BMJ Open.

[83] Anderson B.J., Hannam J.A. (2019). A target concentration strategy to determine ibuprofen dosing in children. Paediatr. Anaesth..

[84] Green R., Webb D., Jeena P.M., Wells M., Butt N., Hangoma J.M., Moodley R.S., Maimin J., Wibbelink M., Mustafa F. (2021). Management of acute fever in children: Consensus recommendations for community and primary healthcare providers in sub-Saharan Africa. Afr. J. Emerg. Med..

[85] Hayashi T., Kitamura K., Hashimoto S., Hotomi M., Kojima H., Kudo F., Maruyama Y., Sawada S., Taiji H., Takahashi G. (2020). Clinical practice guidelines for the diagnosis and management of acute otitis media in children-2018 update. Auris Nasus Larynx.

[86] Doria M., Careddu D., Iorio R., Verrotti A., Chiappini E., Barbero G.M., Ceschin F., Dell’Era L., Fabiano V., Mencacci M. (2021). Paracetamol and ibuprofen in the treatment of fever and acute mild-moderate pain in children: Italian experts’ consensus statements. Children.

[87] Doniec Z., Jackowska T., Sybilski A., Woroń J., Mastalerz-Migas A. (2021). FEVER in children—Recommendations for primary care, doctors: FEVER COMPASS. Fam. Med. Prim. Care Rev..

[88] Hunter L.J., Wood D.M., Dargan P.I. (2011). The patterns of toxicity and management of acute nonsteroidal anti-inflammatory drug (NSAID) overdose. Open Access Emerg. Med..

[89] Hall A.H., Smolinske SCConrad F.L., Wruk K.M., Kulig KWDwelle T.L., Rumack B.H. (1986). Ibuprofen overdose: 126 cases. Ann. Emerg. Med..

[90] Perrott D.A., Piira T., Goodenough B., Champion G.D. (2004). Efficacy and Safety of Acetaminophen vs Ibuprofen for Treating Children’s Pain or Fever: A Meta-analysis. Arch. Pediatr. Adolesc. Med..

[91] Tan E., Braithwaite I., McKinlay C.J.D., Dalziel S.R. (2020). Comparison of Acetaminophen (Paracetamol) With Ibuprofen for Treatment of Fever or Pain in Children Younger Than 2 Years: A Systematic Review and Meta-analysis. JAMA Netw. Open.

[92] Poonai N., Bhullar G., Lin K., Papini A., Mainprize D., Howard J., Teefy J., Bale M., Langford C., Lim R. (2014). Oral administration of morphine versus ibuprofen to manage postfracture pain in children: A randomized trial. CMAJ.

[93] Moss J.R., Watcha M.F., Bendel L.P., McCarthy D.L., Witham S.L., Glover C.D. (2014). A multicenter, randomized, double-blind placebo-controlled, single dose trial of the safety and efficacy of intravenous ibuprofen for treatment of pain in pediatric patients undergoing tonsillectomy. Paediatr. Anaesth..

[94] Smith C., Goldman R.D. (2012). Alternating acetaminophen and ibuprofen for pain in children. Can. Fam. Physician.

[95] Cooney M.F. (2021). Pain Management in Children: NSAID Use in the Perioperative and Emergency Department Settings. Paediatr. Drugs.

[96] Ziesenitz V.C., Welzel T., van Dyk M., Saur P., Gorenflo M., van den Anker J.N. (2022). Efficacy and Safety of NSAIDs in Infants: A Comprehensive Review of the Literature of the Past 20 Years. Paediatr. Drugs.

[97] Mulita F., Karpetas G., Liolis E., Vailas M., Tchabashvili L., Maroulis I. (2021). Comparison of analgesic efficacy of acetaminophen monotherapy versus acetaminophen combinations with either pethidine or parecoxib in patients undergoing laparoscopic cholecystectomy: A randomized prospective study. Med. Glas..

[98] Rutten K., Schröder W., Christoph T., Koch T., Tzschentke T.M. (2018). Selectivity profiling of NOP, MOP, DOP and KOP receptor antagonists in the rat spinal nerve ligation model of mononeuropathic pain. Eur. J. Pharmacol..

[99] Pathan H., Williams J. (2012). Basic opioid pharmacology: An update. Br. J. Pain.

[100] Lambert D.G. (2023). Opioids and opioid receptors; understanding pharmacological mechanisms as a key to therapeutic advances and mitigation of the misuse crisis. Brit. J. Anaesth. Open.

[101] Herzig S.J., Stefan M.S., Pekow P.S., Shieh M.S., Soares W., Raghunathan K., Lindenauer P.K. (2020). Risk Factors for Severe Opioid-Related Adverse Events in a National Cohort of Medical Hospitalizations. J. Gen. Intern. Med..

[102] Lynn A.M., Nespeca M.K., Opheim K.E., Slattery J.T. (1993). Respiratory effects of intravenous morphine infusions in neonates, infants, and children after cardiac surgery. Anesth. Analg..

[103] Lynn A.M., Nespeca M.K., Bratton S.L., Shen D.D. (2000). Intravenous morphine in postoperative infants: Intermittent bolus dosing versus targeted continuous infusions. Pain.

[104] Duedahl T.H., Hansen E.H. (2007). A qualitative systematic review of morphine treatment in children with postoperative pain. Paediatr. Anaesth..

[105] Wong C., Lau E., Palozzi L., Campbell F. (2012). Pain management in children: Part 2—A transition from codeine to morphine for moderate to severe pain in children. Can. Pharm. J..

[106] Kirchheiner J., Henckel H.B., Franke L., Meineke I., Tzvetkov M., Uebelhack R., Roots I., Brockmöller J. (2005). Impact of the CYP2D6 ultra-rapid metabolizer genotype on doxepin pharmacokinetics and serotonin in platelets. Pharmacogenet. Genom..

[107] Kirchheiner J., Henckel H.B., Meineke I., Roots I., Brockmoller J. (2004). Impact of the CYP2D6 ultrarapid metabolizer genotype on mirtazapine pharmacokinetics and adverse events in healthy volunteers. J. Clin. Psychopharmacol..

[108] Gasche Y., Daali Y., Fathi M., Chiappe A., Cottini S., Dayer P., Desmeules J. (2004). Codeine intoxication associated with ultrarapid CYP2D6 metabolism. N. Engl. J. Med..

[109] Dean L., Kane M., Pratt V.M., Scott S.A., Pirmohamed M., Esquivel B., Kattman B.L., Malheiro A.J. (2012). Codeine Therapy and CYP2D6 Genotype. Medical Genetics Summaries [Internet].

[110] Aynacioglu A.S., Sachse C., Bozkurt A., Kortunay S., Nacak M., Schroder T., Roots I., Brockmöller J. (1999). Low frequency of defective alleles of cytochrome P450 enzymes 2C19 and 2D6 in the Turkish population. Clin. Pharmacol. Ther..

[111] McLellan R.A., Oscarson M., Seidegard J., Evans D.A., Ingelman-Sundberg M. (1997). Frequent occurrence of CYP2D6 gene duplication in Saudi Arabians. Pharmacogenetics.

[112] Cartabuke R.S., Tobias J.D., Taghon T., Rice J. (2014). Current practices regarding codeine administration among pediatricians and pediatric subspecialists. Clin. Pediatr..

[113] Tobias J.D., Green T.P., Coté C.J. (2016). Section on Anesthesiology and Pain Medicine; Committee on Drugs. Codeine: Time to Say “No”. Pediatrics.

[114] European Medicines Agency Restrictions on Use of Codeine for Pain Relief in Children—CMDh Endorses PRAC Recommendation. https://www.ema.europa.eu/en/documents/referral/codeine-article-31-referral-restrictions-use-codeine-pain-relief-children-cmdh-endorses-prac_en.pdf.

[115] US Food and Drug Administration FDA Drug Safety Communication: Codeine Use in Certain Children after Tonsillectomy and/or Adenoidectomy May Lead to Rare, but Life-Threatening Adverse Events or Death in Children Because It Was though That It Could be Equally Effective with a Lower Risk of Severe Adverse Events. http://www.fda.gov/Drugs/DrugSafety/ucm313631.htm.

[116] European Medicines Agency Scientific Conclusions and Grounds for the Variation to the Terms of the Marketing Authorisation(s). https://www.ema.europa.eu/en/documents/psusa/tramadol-cmdh-scientific-conclusions-grounds-variation-amendments-product-information-timetable/00003002/201705_en.pdf.

[117] Renny M.H., Jent V., Townsend T., Cerdá M. (2022). Impact of the 2017 FDA Drug Safety Communication on Codeine and Tramadol Dispensing to Children. Pediatrics.

[118] US Food and Drug Administration FDA Drug Safety Communication: FDA Restricts Use of Prescription Codeine Pain and Cough Medicines and Tramadol Pain Medicines in Children; Recommends Against use in Breastfeeding Women. https://www.fda.gov/downloads/Drugs/DrugSafety/UCM553814.pdf.

[119] Rodieux F., Vutskits L., Posfay-Barbe K.M., Habre W., Piguet V., Desmeules J.A., Samer C.F. (2018). When the Safe Alternative Is Not That Safe: Tramadol Prescribing in Children. Front. Pharmacol..

[120] Beakley B.D., Kaye A.M., Kaye A.D. (2015). Tramadol, Pharmacology, Side Effects, and Serotonin Syndrome: A Review. Pain Physician.

[121] Lee C.R., McTavish D., Sorkin E.M. (1993). Tramadol. A preliminary review of its pharmacodynamic and pharmacokinetic properties, and therapeutic potential in acute and chronic pain states. Drugs.

[122] Rose J.B., Finkel J.C., Arquedas-Mohs A., Himelstein B.P., Schreiner M., Medve R.A. (2003). Oral tramadol for the treatment of pain of 7–30 days’ duration in children. Anesth. Analg..

[123] Neri E., Maestro A., Minen F., Montico M., Ronfani L., Zanon D., Favret A., Messi G., Barbi E. (2013). Sublingual ketorolac versus sublingual tramadol for moderate to severe post-traumatic bone pain in children: A double-blind, randomised, controlled trial. Arch. Dis. Child..

[124] Uysal H.Y., Takmaz S.A., Yaman F., Baltaci B., Başar H. (2011). The efficacy of intravenous paracetamol versus tramadol for postoperative analgesia after adenotonsillectomy in children. J. Clin. Anesth..

[125] Li S., Xiong H., Jia Y., Li Z., Chen Y., Zhong L., Liu F., Qu S., Du Z., Wang Y. (2023). Oxycodone vs. tramadol in postoperative parent-controlled intravenous analgesia in children: A prospective, randomized, double-blinded, multiple-center clinical trial. BMC Anesthesiol..

[126] Schnabel A., Reichl S.U., Meyer-Frießem C., Zahn P.K., Pogatzki-Zahn E. (2015). Tramadol for postoperative pain treatment in children. Cochrane Database Syst. Rev..

[127] Borland M., Jacobs I., King B., O’Brien D. (2007). A randomised controlled trial comparing intranasal fentanyl to intravenous morphine for managing acute pain in the emergency department. Ann. Emerg. Med..

[128] Younge P.A., Nicol M.F., Kendall J.M., Harrington A.P. (1999). A prospective randomised pilot comparison of intranasal fentanyl and intramuscular morphine for analgesia in children presenting to the emergency department with clinical fractures. Emerg. Med..

[129] Manjushree R., Lahiri A., Ghosh B.R., Laha A., Handa K. (2002). Intranasal fentanyl provides adequate postoperative analgesia in paediatric patients. Can. J. Anaesth..

[130] Borland M., Jacobs I., Geelhoed G. (2002). Intranasal Fentanyl reduces acute pain in children in the emergency department: A safety and efficacy study. Paediatr. Emergy Med..

[131] Serra S., Spampinato M.D., Riccardi A., Guarino M., Pavasini R., Fabbri A., De Iaco F. (2023). Intranasal Fentanyl for Acute Pain Management in Children, Adults and Elderly Patients in the Prehospital Emergency Service and in the Emergency Department: A Systematic Review. J. Clin. Med..

[132] European Medicines Agency Durogesic and Associated Names. https://www.ema.europa.eu/en/medicines/human/referrals/durogesic-associated-names.

[133] Taylor K.P., Singh K., Goyal A. (2023). Fentanyl Transdermal. [Updated 2022 Nov 9]. StatPearls [Internet].

[134] Armenian P., Vo K.T., Barr-Walker J., Lynch K.L. (2018). Fentanyl, fentanyl analogs and novel synthetic opioids: A comprehensive review. Neuropharmacology.

[135] Choi L., Ferrell B.A., Vasilevskis E.E., Pandharipande P.P., Heltsley R., Ely E.W., Stein C.M., Girard T.D. (2016). Population Pharmacokinetics of Fentanyl in the Critically Ill. Crit. Care Med..

[136] Nozari A., Akeju O., Mirzakhani H., Eskandar E., Ma Z., Hossain M.A., Wang Q., Greenblatt D.J., Martyn J.A.J. (2019). Prolonged therapy with the anticonvulsant carbamazepine leads to increased plasma clearance of fentanyl. J. Pharm. Pharmacol..

[137] Poonai N., Creene C., Dobrowlanski A., Geda R., Hartling L., Ali S., Bhatt M., Trottier E.D., Sabhaney V., O’Hearn K. (2023). Inhaled nitrous oxide for painful procedures in children and youth: A systematic review and meta-analysis. CJEM.

[138] Heinrich M., Menzel C., Hoffmann F., Berger M., von Schweinitz D. (2015). Self-administered procedural analgesia using nitrous oxide/oxygen (50, 50) in the pediatric surgery emergency room: Effectiveness and limitations. Eur. J. Pediatr. Surg..

[139] Zier J.L., Liu M. (2011). Safety of high-concentration nitrous oxide by nasal mask for pediatric procedural sedation: Experience with 7802 cases. Pediatr. Emerg. Care.

[140] Gupta N., Gupta A., Narayanan MR V. (2022). Current status of nitrous oxide use in pediatric patients. World J. Clin. Pediatr..

[141] Green S.M., Roback M.G., Kennedy R.M., Krauss B. (2011). Clinical practice guideline for emergency department ketamine dissociative sedation: 2011 update. Ann. Emerg. Med..

[142] Lee E.N., Lee J.H. (2016). The Effects of Low-Dose Ketamine on Acute Pain in an Emergency Setting: A Systematic Review and Meta-Analysis. PLoS ONE.

[143] Alanazi E. (2022). The Effectiveness of Ketamine Compared to Opioid Analgesics for management of acute pain in Children in The Emergency Department: Systematic Review. Am. J. Emerg. Med..

[144] Zhao Y., He J., Yu N., Jia C., Wang S. (2020). Mechanisms of Dexmedetomidine in Neuropathic Pain. Front. Neurosci..

[145] Mason K.P., Lerman J. (2011). Review article: Dexmedetomidine in children: Current knowledge and future applications. Anesth. Analg..

[146] Mahmoud M., Barbi E., Mason K.P. (2020). Dexmedetomidine: What’s New for Pediatrics? A Narrative Review. J. Clin. Med..

[147] Sottas C.E., Anderson B.J. (2017). Dexmedetomidine: The new all-in-one drug in paediatric anaesthesia?. Curr. Opin. Anesthesiol..

[148] Raffaeli G., Orenti A., Gambino M., Peves Rios W., Bosis S., Bianchini S., Tagliabue C., Esposito S. (2016). Fever and Pain Management in Childhood: Healthcare Providers’ and Parents’ Adherence to Current Recommendations. Int. J. Environ. Res. Public Health.

[149] Chiappini E., Principi N., Longhi R., Tovo P.A., Becherucci P., Bonsignori F., Esposito S., Festini F., Galli L., Lucchesi B. (2009). Management of fever in children: Summary of the Italian Pediatric Society guidelines. Clin. Ther..

